# The expression pattern and prognostic relevance of p120-catenin, COL4A2 and SOX10 in glioma

**DOI:** 10.3389/fonc.2026.1769786

**Published:** 2026-02-05

**Authors:** Claudia Alexandra Dumitru, David Markus Andreas Schmidt, Belal Neyazi, Klaus-Peter Stein, Ali Rashidi, Ulf Dietrich Kahlert, Christian Mawrin, Ludwig Wilkens, Ibrahim Erol Sandalcioglu

**Affiliations:** 1 Department of Neurosurgery, Otto-von-Guericke University, Magdeburg, Germany; 2 Molecular and Experimental Surgery, Department of General, Visceral, Vascular and Transplantation Surgery, Otto-von-Guericke University, Magdeburg, Germany; 3 Department of Neuropathology, Otto-von-Guericke University, Magdeburg, Germany; 4 Department of Pathology, Otto-von-Guericke University, Magdeburg, Germany; 5 Department of Pathology, KRH Nordstadt Hospital, Hannover, Germany

**Keywords:** biomarkers, glioblastoma, glioma, outcome, prognosis, sex-related differences

## Abstract

**Background:**

p120-catenin, COL4A2 and SOX10 are emerging as modulators of glioma pathophysiology and progression. This study aimed to characterize the expression pattern of these markers in glioma tissues with different degrees of malignancy, and tested their prognostic value for the outcome of glioblastoma IDH wild-type (GBM IDH^wt^) patients, with an additional focus on potential sex-related differences.

**Methods:**

All markers were assessed by immunohistochemistry in tissue microarrays prepared from healthy brain (n=38), astrocytoma grade 2 (n=24), astrocytoma grade 3 (n=22), and GBM IDH^wt^ (n=204) samples. Correlation analyses were performed using Spearman’s Rho, and survival analyses (5-year overall survival and 1-year progression-free survival) were performed using Kaplan-Meier curves, log-rank test and multivariate proportional hazard models.

**Results:**

The levels of p120-catenin significantly increased with the degree of glioma malignancy (p<0.001; Rho=0.599), while the opposite was observed for COL4A2 (p<0.001, Rho=-0.387) and SOX10 (p<0.001; Rho=-0.293). High levels of p120-catenin significantly associated with and predicted the poor overall survival of GBM IDH^wt^ patients (HR = 1.861, CI = 1.303-2.658, p<0.001) both male (HR = 1.709, CI = 1.077-2.713, p=0.023) and female (HR = 2.141, CI = 1.138-4.028, p=0.018). Conversely, low levels of SOX10 associated with and predicted the poor overall survival of GBM IDH^wt^ patients (HR = 1.552, CI = 1.025-2.352, p=0.038). Interestingly, SOX10 was an independent prognostic factor only in female patients (HR = 2.842, CI = 1.241-6.511, p=0.014). Regarding progression-free survival, p120-catenin was a significant prognostic factor in the whole cohort of GBM IDH^wt^ patients (HR = 2.542; CI = 1.499-4.312; p<0.001) and in the male patients (HR = 2.431; CI = 1.222-4.836; p=0.011), while SOX10 did not predict the progression-free survival in any group of patients. For COL4A2, we found no significant associations with the patients’ outcome, irrespective of sex.

**Conclusions:**

p120-catenin is a potential tumor-promoting factor in glioma, and a prognostic marker in GBM. In contrast, COL4A2 and SOX10 appear to act as tumor suppressors in glioma pathophysiology. SOX10 may additionally be a valuable prognostic marker in female GBM patients.

## Introduction

Gliomas are the most common malignant primary tumors of the central nervous system (CNS) in adults. Histo-pathologically, gliomas are categorized based on cellular etiology (astrocytic, oligodendrocytic, ependymal), and on their degree of malignancy. Almost 80% of all gliomas are astrocytic tumors, out of which 7.2% are diffuse astrocytoma WHO grade 2, 4.8% anaplastic astrocytoma WHO grade 3, and 61.7% glioblastoma (GBM) WHO grade 4 (1). Since the fifth edition of the WHO classification of CNS tumors from 2021, only astrocytic tumors WHO grade 4 with an IDH wild-type (IDH^wt^) phenotype are considered to be GBM (2). Although the median survival times for different glioma entities vary across studies, the latest CBTRUS report indicates a median survival of 61 and 22 months for patients with astrocytoma grade 2 and 3, respectively (1). GBM patients have a very short median survival of only 8 months (1), despite undergoing an aggressive therapeutic regimen consisting of maximally safe tumor resection, radiotherapy, and chemotherapy with alkylating agents (3). Newer multimodal therapeutic strategies directed against receptor tyrosine kinases (RTKs), or modulating the stem cell, apoptosis and cell cycle/DNA repair pathways (for a systematic review see (4)) have also met with only limited success. Thus, it remains necessary to identify novel cellular and molecular factors that are involved in glioma progression, and may serve as targets for therapy in this type of cancer.

P120-catenin (p120, catenin delta-1), which is encoded by the *CTNND1* gene, is a structural protein with critical functions in cellular adhesion. In physiological processes, p120-catenin interacts with E-cadherin to promote cell-cell adhesion, but also facilitates the stabilization, bundling, and tethering of microtubules at the adherens junctions (for a recent review see (5)). In cancer, p120-catenin can act as both tumor suppressor and tumor promoter. Specifically, p120-catenin is downregulated in several types of cancer, such as colon, stomach, breast, lung or pancreas (reviewed in (5)), and low protein levels of p120-catenin associate with the poor survival of patients with intrahepatic cholangiocarcinoma (6) and invasive ductal breast cancer (7). In contrast, other studies found that p120-catenin enhanced the migration/invasion of tumor cells, thereby promoting tumor progression in colorectal, breast, and ovarian cancer (8–10). Recent evidence indicates that p120-catenin has tumor-promoting functions also in glioma, since it is overexpressed in these tumors compared to healthy brain tissues, and facilitates the migration/invasion and proliferation of the glioma cells (11–13).

COL4A2 -the alpha 2 subunit of Collagen IV- is a protein highly conserved among species and ubiquitously expressed in basement membranes. Mutations in the *COL4A2* gene have been linked to a variety of medical conditions, in particular cerebrovascular diseases and porencephaly, but were also shown to cause ocular, renal and muscular defects (reviewed in (14)). Most previous studies observed an upregulation of *COL4A2* gene expression in tumor tissues, including gliomas (15–17), which indicates a potential tumor-promoting role of this factor. However, others reported that COL4A2 was downregulated in tumor cells, and that low protein levels of COL4A2 associated with high TNM stage, as well as with the poor outcome of cancer patients (18–20).

SOX10 is a transcription factor crucial for the differentiation, migration and maintenance of tissues derived from the neural crest (reviewed in (21)). Like p120-catenin, SOX10 can act as both tumor promoter and suppressor in human cancers. For instance, SOX10 protein is overexpressed in nasopharyngeal carcinoma and correlates with high TNM stage as well as with the poor outcome of these patients (22). Similar observations were made in bladder carcinoma, where additional functional studies showed that *SOX10* knock-down significantly inhibited the proliferation and the migration/invasion of the tumor cells (23). In contrast, SOX10 protein expression was downregulated or even completely absent in some cancers, such as gastrointestinal mesenchymal tumors or melanoma resistant to immune checkpoint inhibitors (24, 25). In glioma, previous exploratory findings indicated that SOX10 protein was downregulated in tumor tissues compared to healthy brain tissues (26), and *SOX10* loss associated with a more aggressive tumor cell phenotype (27, 28).

The above-mentioned studies support a potential role of p120-catenin, COL4A2 and SOX10 in glioma/GBM pathophysiology. However, detailed studies on patients with strictly defined glioma entities, which additionally involved multivariate proportional hazard models, are missing thus far. In this study, we aimed to assess 1) the expression of the markers in healthy brain tissues and gliomas with different degrees of malignancy, 2) the association between marker expression and the outcome of the patients with GBM IDH^wt^ tumors and 3) the prognostic value of the markers in these patients using multivariate models. Furthermore, all markers were tested for potential sex-related differences regarding their association with the patients’ outcome and prognosis.

## Materials and methods

### Study subjects

This study included adult patients with newly diagnosed and histologically confirmed astrocytoma WHO grades 2 and 3, as well as grade 4 GBM IDH^wt^ tumors. The astrocytoma grade 2 group consisted of 24 patients, grade 3 of 22 patients, and the GBM IDH^wt^ group included 204 patients. The median ages of these groups were 43, 49.5 and 67 years, respectively. The patients were treated at the Department of Neurosurgery, KRH Nordstadt Hospital Hannover between 2004 and 2014, and the corresponding formalin-fixed paraffin-embedded (FFPE) tissues were archived at the Department of Pathology of the same institution. The ethics committee of the Medical School Hannover approved this study (No. 6864, 2015) and additionally waivered the need for informed written consent.

### Study design

The follow-up data regarding overall- and progression-free survival was retrieved for each patient. Furthermore, we retrieved the relevant clinical characteristics of the patients, including sex, Karnofsky Performance Scale (KPS), therapy and extent of surgical resection, but also molecular characteristics, such as the MGMT and IDH status (see **Table 1**). The FFPE tissues were processed into tissue microarrays (TMAs), stained and digitalized as described in our previous studies (29, 30). Where available, cores from different regions of the tumor were included in the TMAs, to cover for potential heterogeneity. Non-malign brain parenchyma (hereafter referred to as “healthy brain tissues”) could also be retrieved from 38 patients, and were subsequently included in the TMAs.

**Table 1:** Clinical characteristics of the patients included in this study.

	Astrocytoma grade 2 (n=24)		Astrocytoma grade 3 (n=22)		GBM IDHwt (n=204)	
	Number	Percentage	Number	Percentage	Number	Percentage
Sex						
female	10	41.7	11	50.0	81	39.7
male	14	58.3	11	50.0	123	60.3
KPS						
10	0	0.0	0	0.0	2	1.0
20	0	0.0	0	0.0	1	0.5
30	1	4.2	1	4.5	2	1.0
40	0	0.0	0	0.0	13	6.4
50	0	0.0	1	4.5	28	13.7
60	0	0.0	3	13.6	43	21.1
70	1	4.2	6	27.3	43	21.1
80	11	45.8	6	27.3	37	18.1
90	9	37.5	5	22.7	26	12.7
100	0	0.0	0	0.0	1	0.5
n.d.	2	8.3	0	0.0	8	3.9
Therapy						
surgery	10	41.7	1	4.5	23	11.3
surgery+RTX	4	16.7	3	13.6	28	13.7
surgery+CTX	1	4.2	3	13.6	4	2.0
surgery+RCTX	7	29.2	15	68.2	140	68.6
n.d.	2	8.3	0	0.0	9	4.4
Resection						
subtotal	6	25.0	7	31.8	120	58.8
total	13	54.2	11	50.0	72	35.3
n.d.	5	20.8	4	18.2	12	5.9
MGMT status						
unmethylated	6	25.0	7	31.8	95	46.6
methylated	11	45.8	12	54.5	94	46.1
n.d.	7	29.2	3	13.6	15	7.4
IDH status						
wild-type	10	41.7	12	54.5	204	100.0
mutated	13	54.2	8	36.4	0	0.0
n.d.	1	4.2	2	9.1	0	0.0

CTX, chemotherapy; KPS, Karnofsky performance scale; n.d., not determinable; RCTX, radio-chemotherapy; RTX, radiotherapy.

### Tissue analysis and scoring system

The TMAs were stained with the following primary antibodies: Cell Marque™ anti-human p120-catenin (MRQ-5) mouse monoclonal antibody (Sigma-Aldrich, Taufkirchen, Germany), anti-human COL4A2 rabbit polyclonal antibody (Novus Biologicals/Bio-Techne, Wiesbaden, Germany) and Cell Marque™ anti-human SOX10 rabbit polyclonal antibody (Sigma-Aldrich, Taufkirchen, Germany). The samples were scored and validated independently by authors C.A.D., D.M.A.S., L.W. and C.M.

**Figure 1:**
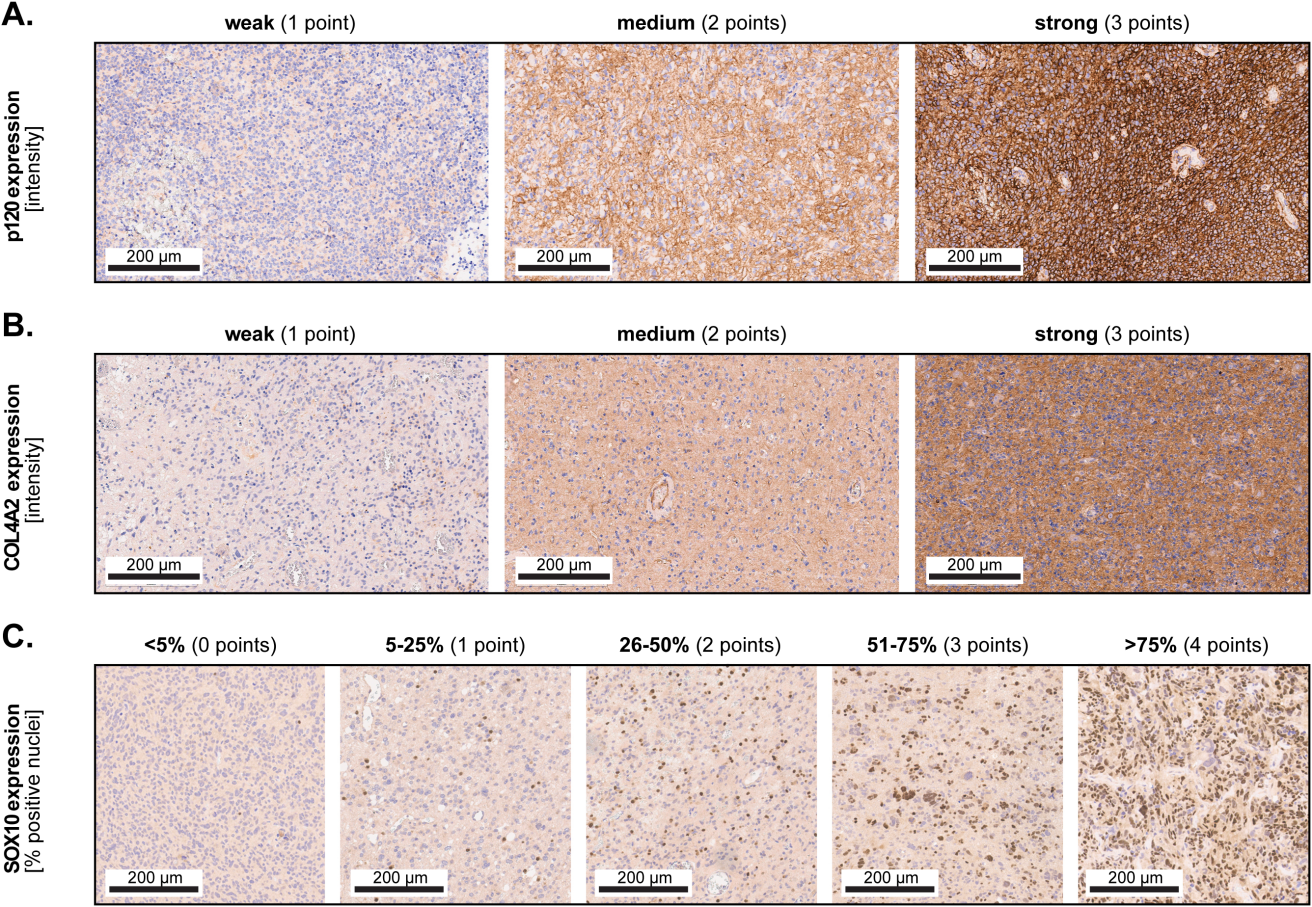
Marker expression and scoring. Representative micrographs showing weak (1 point), medium (2 points) and strong (3 points) expression of **(A)** p120-catenin and **(B)** COL4A2. Subsequently, the H-score was calculated based on the formula (1 x X) + (2 x Y) + (3 x Z), where X + Y + Z = 100% of the total analyzed area. **(C)** The 5-tier score for SOX10 according to the percentage of positive cells.

P120-catenin was localized both in the cytoplasm and in the plasma membrane of the cells, while COL4A2 displayed mainly a cytoplasmic subcellular localization. Both markers were expressed with varying intensities, which we categorized as ‘weak’ (1 point), ‘medium’ (2 points) and ‘strong’ (3 points) (**Figures 1A, B**). We also observed a heterogeneous distribution pattern of the expression intensity in many samples. Therefore, the final expression levels for these markers were calculated using the H-score formula: (1 x X) + (2 x Y) + (3 x Z), where X + Y + Z = 100% of the tumor area. In contrast, SOX10 was localized exclusively in the cell nuclei, and did not exhibit a great variance regarding expression intensity. Consequently, SOX10 was assessed only based on the percentage of positive cells using a 5-tier score: <5% (0 points), 5-25% (1 point), 26-50% (2 points), 51-75% (3 points) and >75% (4 points) (**Figure 1C**). To account for potential heterogeneity, we analyzed 6 non-overlapping microscopic fields per TMA spot at 200-fold magnification, and subsequently averaged the values for each sample. For samples where only little tumor tissue was available (due to large necrotic areas or technical issues), we analyzed 4 microscopic fields per sample.

### Statistical analysis

The data was analyzed with the SPSS statistics software version 29.0.2.0 (IBM Corporation, Armonk, NY, USA). The expression levels of the markers in relation to the degree of tumor malignancy was visualized using box-whisker plots, and the statistical analysis was performed with the Spearman’s rank test (Spearman’s Rho). The association between marker expression and the survival of the GBM IDH^wt^ patients (5-year for overall survival and 1-year for progression-free survival) was initially assessed using Kaplan-Meier curves and log-rank univariate analysis. The prognostic significance of the markers was subsequently tested by multivariate analysis using Cox proportional hazard regression models adjusted for age, KPS, extent of surgical resection, therapy and MGMT methylation status. These analyses were performed for the entire GBM patient cohort, but also separately for male and female patients. Additionally, interaction testing was performed in the entire GBM cohort by calculating the interaction term SOX10*Sex, which was subsequently included in the Cox regression models. The level of significance was set at p ≤ 0.05 in all statistical analyses.

## Results

**Figure 2:**
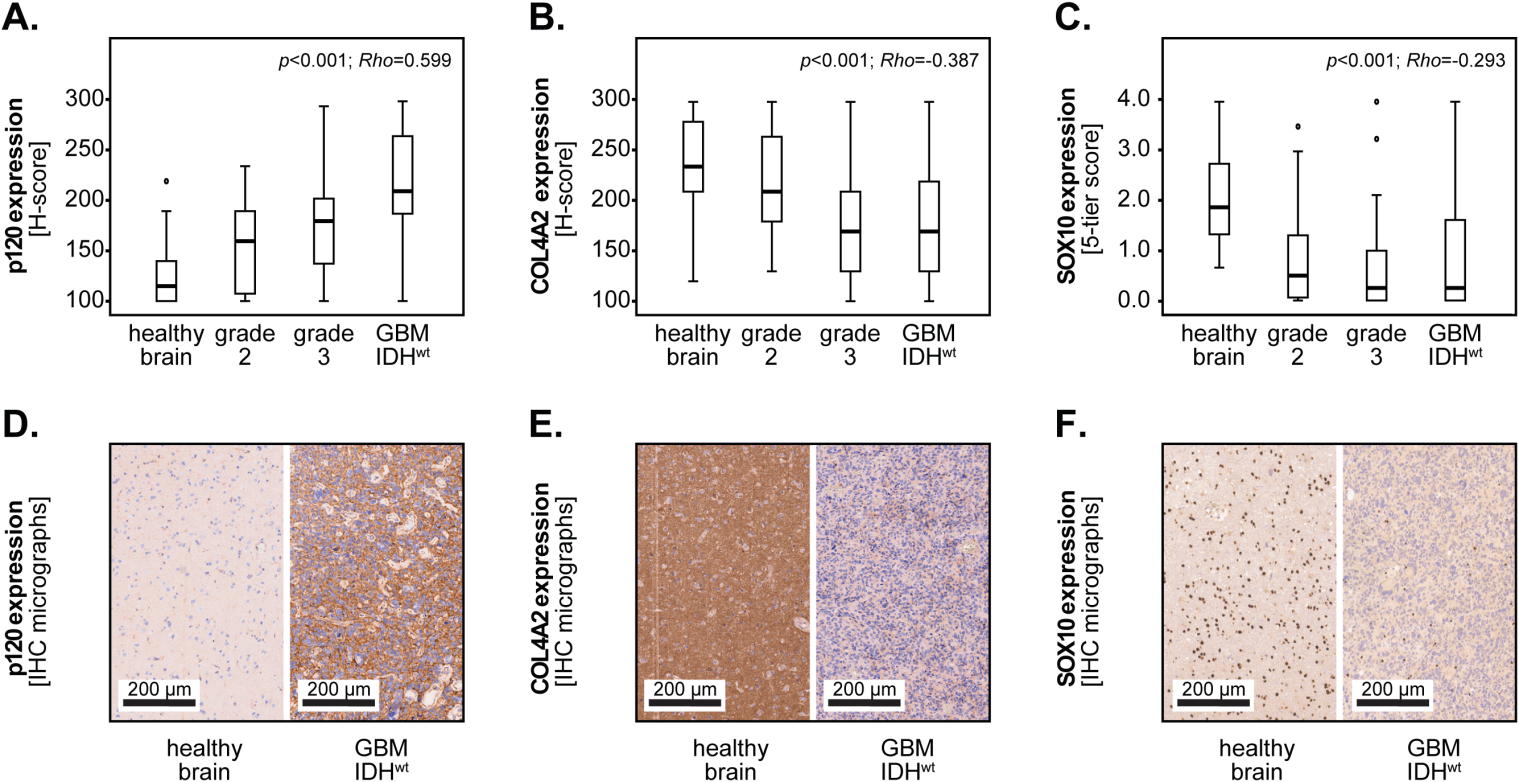
Marker expression according to the degree of malignancy. Expression of **(A)** p120-catenin, **(B)** COL4A2 and **(C)** SOX10 in healthy brain, astrocytoma grade 2, astrocytoma grade 3, and GBM IDH^wt^ tissues. The medians are shown as bold lines and the percentiles (25^th^ and 75^th^) as vertical boxes with error bars. The outliers are indicated by circles. Statistical analysis was performed with Spearman’s rank test. The p-values and the Rho correlation coefficients are shown in the upper right corner of the plots. Representative micrographs depicting the expression of **(D)** p120-catenin, **(E)** COL4A2 and **(F)** SOX10 in healthy brain versus GBM IDH^wt^ tissues.

In the first set of studies, we analyzed the expression of the three biomarker candidates in relation to the tumor grade. From 38 patients, we were also able to analyze the healthy brain tissue adjacent to the tumor. The results showed that the expression of p120-catenin was weak in the healthy brain tissues (median H-score=115) and gradually increased with the tumor grade (grade 2 = 160; grade 3 = 180; GBM = 210). Statistical analysis using Spearman’s rank test indicated a highly significant positive correlation between p120-catenin and the tumor grade (p<0.001, Rho=0.599, Spearman) (**Figure 2A**). In contrast, the expression of COL4A2 was high in the healthy brain tissues (median=235), decreased slightly in grade 2 tumors (median=210), and was lowest in the high-grade gliomas (grade 3 = 170; GBM = 170). Spearman’s rank test confirmed a significant inverse correlation between COL4A2 and the tumor grade (p<0.001; Rho=-0.387, Spearman) (**Figure 2B**). SOX10 was also highly expressed in the healthy brain tissues (median 5-tier score=1.87) and decreased with the tumor grade (grade 2 = 0.50; grade 3 = 0.25; GBM = 0.25), thereby showing a significant inverse correlation (p<0.001, Rho=-0.293, Spearman) (**Figure 2C**). Representative micrographs depicting the expression of the biomarker candidates in healthy brain versus GBM tissues are presented in **Figures 2D-F**.

**Figure 3:**
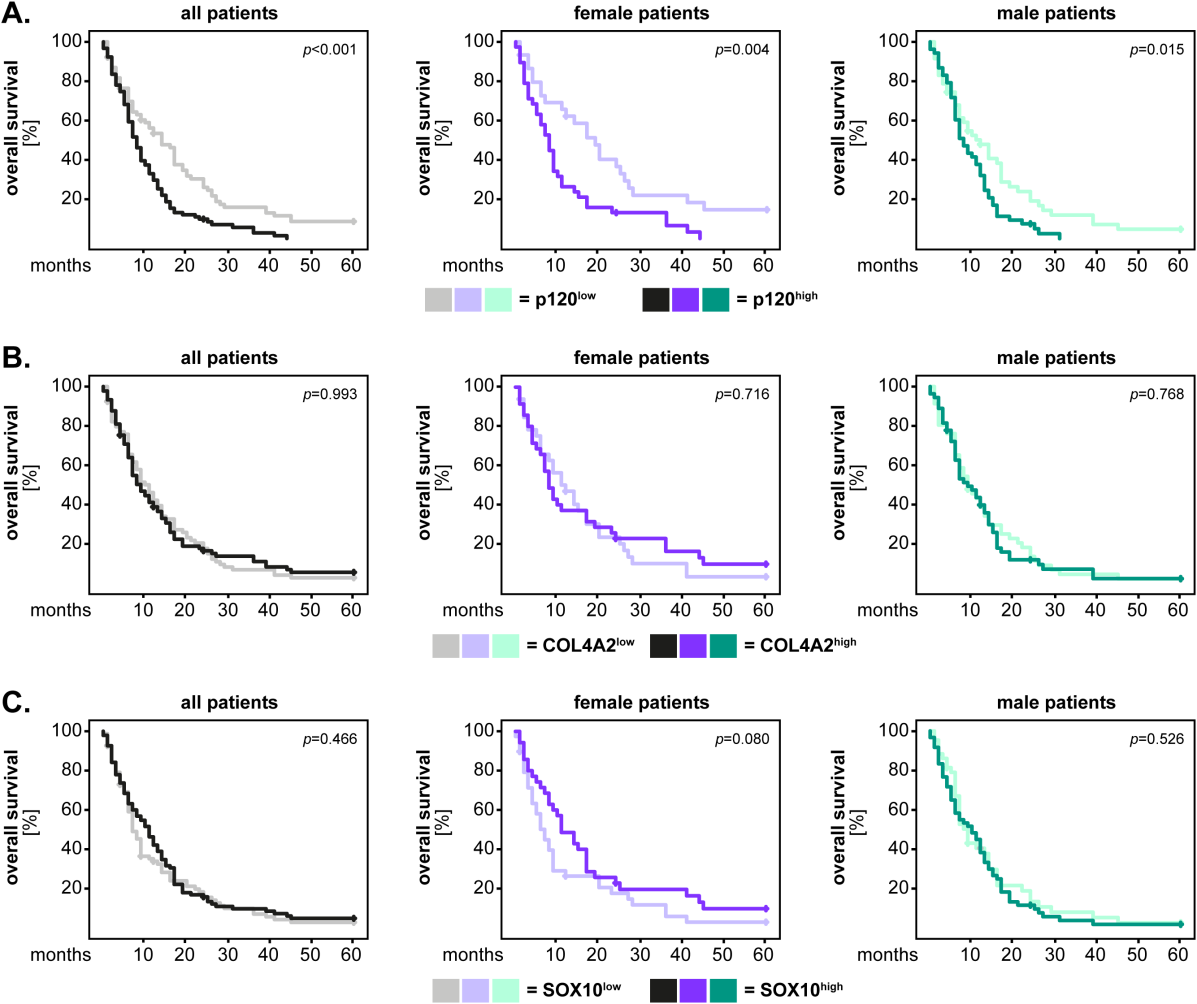
Marker expression (median-split cut-off) and the univariate analysis of overall survival in GBM IDH^wt^ patients. The expression levels of **(A)** p120-catenin, **(B)** COL4A2 and **(C)** SOX10 were dichotomized into “low” and “high” according to the median-split method. The 5-year survival curves were generated with the Kaplan-Meier method, and the statistical analysis was performed with the log-rank test. The p-values are indicated in the upper right corner of the plots. The analyses were carried out for the whole patient cohort (grayscale), as well as separately for female (purple) and male (green) patients. The groups with low levels of the markers are depicted in light colors, while the groups with high expression of the markers are depicted in dark colors.

**Figure 4:**
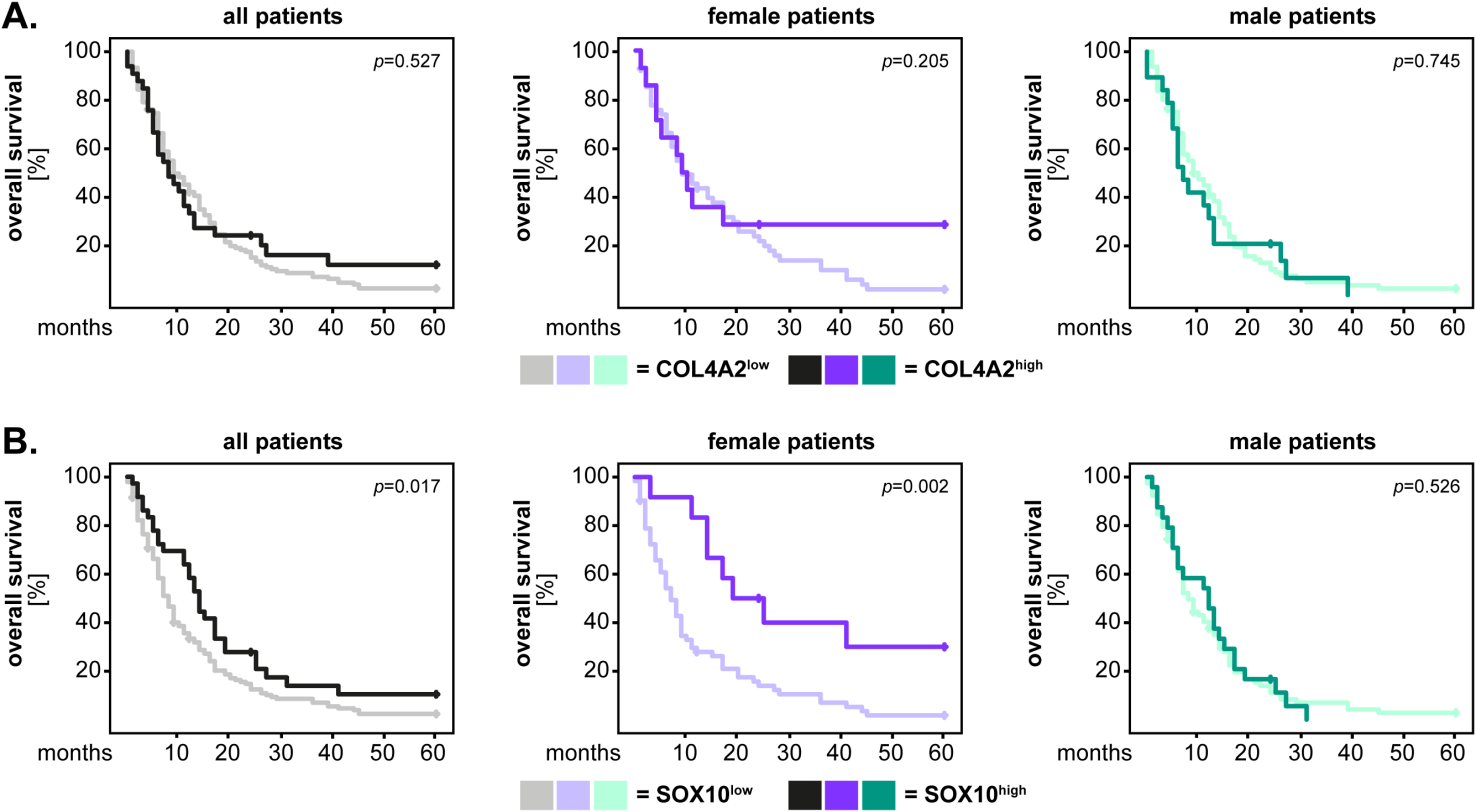
COL4A2 and SOX10 expression (new cut-off) and the univariate analysis of overall survival in GBM IDH^wt^ patients. The expression levels of **(A)** COL4A2 and **(B)** SOX10 were dichotomized into “low” and “high” according to the median values of the healthy brain tissues. The 5-year survival curves were generated with the Kaplan-Meier method, and the statistical analysis was performed with the log-rank test. The p-values are indicated in the upper right corner of the plots. The analyses were carried out for the whole patient cohort (grayscale), as well as separately for female (purple) and male (green) patients. The groups with low levels of the markers are depicted in light colors, while the groups with high expression of the markers are depicted in dark colors.

Next, we investigated the potential association between marker expression and the patients’ outcome. These and all the following studies were performed on the GBM IDH^wt^ cohort, which was the only one sufficiently large to permit a robust statistical analysis. To this end, the expression levels of the markers were first dichotomized according to the median-split method into “marker^low^” and “marker^high^” groups. The data was visualized by Kaplan-Meier survival curves (5-year for overall survival and 1-year for progression-free survival) and the statistical analysis was performed with the log-rank test. These analyzes were carried out for the entire GBM IDH^wt^ cohort (all patients), but also separately for female and male patients, in order to identify potential sex-related differences of the markers. The results showed that the GBM IDH^wt^ patients with high p120-catenin levels had a significantly shorter overall survival compared to the patients with low levels of p120-catenin (p<0.001, log-rank). This pattern was present in both female (p=0.004, log-rank) and male (p=0.015, log-rank) patients (**Figure 3A**). In contrast, no significant differences were observed regarding the overall survival of patients with high and low levels of COL4A2 (**Figure 3B**) and SOX10 (**Figure 3C**), respectively. We therefore sought to determine whether the dichotomization of the latter markers using another cut-off might yield different results. To this end, we selected the median values of the healthy brain tissues as a new cut-off. The rationale behind this selection was that the healthy brain samples expressed physiological levels of COL4A2 and SOX10, while a loss of these markers (as seen in many GBM tissues) might be associated with a pathological process. For COL4A2, there was no significant association with the patients’ overall survival, even with the new cut-off (**Figure 4A**). However, patients with low levels of SOX10 had now a significantly shorter overall survival compared to SOX10^high^ patients (p=0.017, log-rank). Interestingly, there was also a clear sex-related difference regarding SOX10, as this pattern was observed in female (p=0.002, log-rank) but not in male (p=0.526, log-rank) patients (**Figure 4B**).

**Figure 5:**
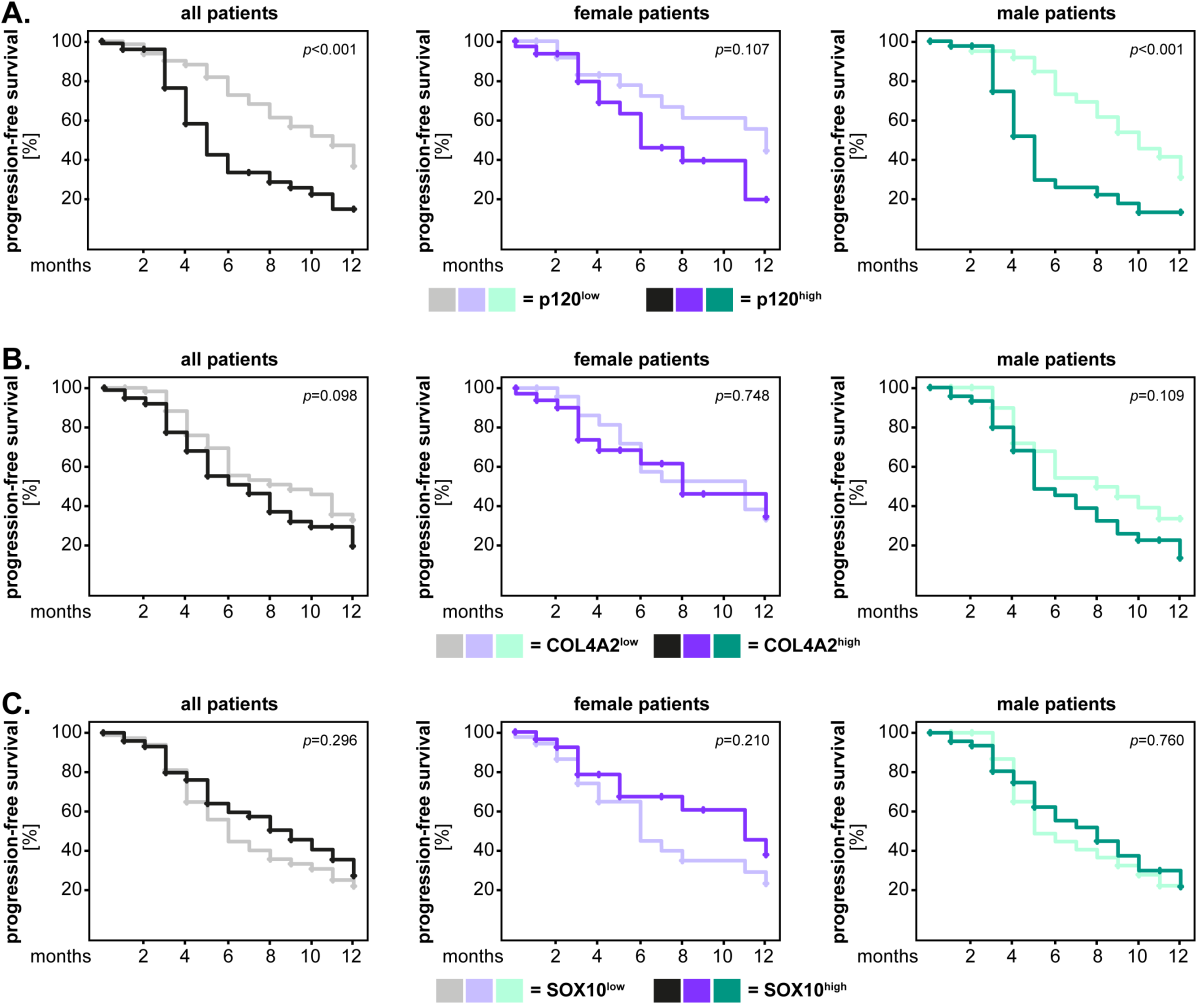
Marker expression (median-split cut-off) and the univariate analysis of progression-free survival in GBM IDH^wt^ patients. The expression levels of **(A)** p120-catenin, **(B)** COL4A2 and **(C)** SOX10 were dichotomized into “low” and “high” according to the median-split method. The 1-year survival curves were generated with the Kaplan-Meier method, and the statistical analysis was performed with the log-rank test. The p-values are indicated in the upper right corner of the plots. The analyses were carried out for the whole patient cohort (grayscale), as well as separately for female (purple) and male (green) patients. The groups with low levels of the markers are depicted in light colors, while the groups with high expression of the markers are depicted in dark colors.

**Figure 6:**
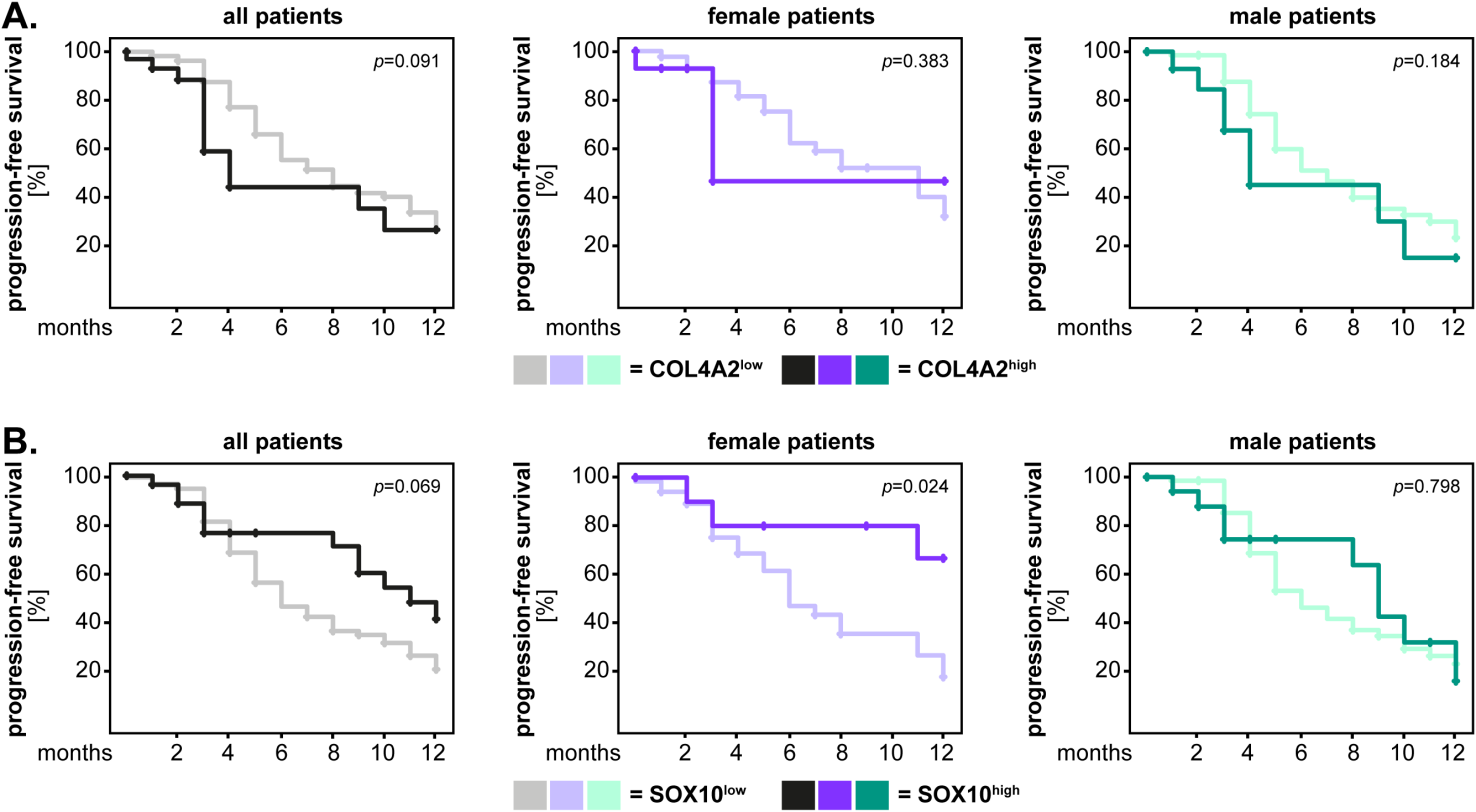
COL4A2 and SOX10 expression (new cut-off) and the univariate analysis of progression-free survival in GBM IDH^wt^ patients. The expression levels of **(A)** COL4A2 and **(B)** SOX10 were dichotomized into “low” and “high” according to the median values of the healthy brain tissues. The 1-year survival curves were generated with the Kaplan-Meier method, and the statistical analysis was performed with the log-rank test. The p-values are indicated in the upper right corner of the plots. The analyses were carried out for the whole patient cohort (grayscale), as well as separately for female (purple) and male (green) patients. The groups with low levels of the markers are depicted in light colors, while the groups with high expression of the markers are depicted in dark colors.

We performed similar analyses regarding the progression-free survival of the GBM IDH^wt^ patients. The results showed that GBM IDH^wt^ patients with high levels of p120-catenin had a significantly shorter progression-free survival compared to the p120^low^ patients (p<0.001, log-rank). Although this pattern was observed in both sexes, the statistical significance was reached only in male patients (p<0.001, log-rank) (**Figure 5A**). For COL4A2 and SOX10 there was no significant association between marker expression and the progression-free survival of the patients (**Figures 5B, C**). Consequently, we tested again the new cut-off of these markers using the median values of the healthy brain tissues. COL4A2 remained not significantly associated with the progression-free survival of GBM IDH^wt^ patients, regardless of sex (**Figure 6A**). However, low levels of SOX10 tended to associate with a shorter progression-free survival in the entire GBM patient cohort (p=0.069, log-rank) and reached statistical significance in the female patients (p=0.024, log-rank) (**Figure 6B**).

In the final set of studies, we employed multivariate Cox regression models to test the prognostic values of the biomarker candidates regarding the patients’ overall and progression-free survival, respectively. These analyzes were carried out only for the markers that had shown statistical significance in the univariate survival analysis, namely p120-catenin (with the median-split cut-off) and SOX10 (with the new cut-off based on the healthy brain tissues’ median). The multivariate models were adjusted for several potential confounders such as age, KPS, extent of surgical resection, therapy, and MGMT methylation status. The results showed that high levels of p120-catenin significantly predicted the shorter overall survival of GBM IDH^wt^ patients, both male and female (**Table 2**). SOX10 was an independent prognostic factor for the overall survival in the female patient cohort, where patients with low levels of SOX10 had an almost three-fold increased risk of death compared to SOX10^high^ patients (**Table 2**). To confirm the sex-specific prognostic effects of SOX10, we carried out further interaction testing in the entire cohort of GBM IDH^wt^ patients. To this end, we calculated the interaction term SOX10*Sex and subsequently performed Cox regression analyses using a basic model (only SOX10, Sex, and SOX10*Sex), as well as a full model, which additionally included the 5 confounders mentioned above. In both models, SOX10*Sex was a significant predictor of poor overall survival (basic model: HR = 3.061, CI = 1.268-7.390, p=0.013; full model: HR = 2.874, CI = 1.125-7.343, p=0.027). For progression-free survival, p120-catenin was an independent prognostic factor in the whole GBM IDH^wt^ cohort and in the male patients, while SOX10 did not reach statistical significance in any group of patients (**Table 3**).

**Table 2:** Multivariate Cox regression analysis of p120-catenin and SOX10 in relation to overall survival in GBM IDH^wt^ patients.

	All patients			Female patients			Male patients		
	HR	CI	*P*-value	HR	CI	*P*-value	HR	CI	*P*-value
**p120^low^**	1			1			1		
**p120^high^**	1.861	1.303-2.658	<0.001	2.141	1.138-4.028	0.018	1.709	1.077-2.713	0.023
age	1.026	1.010-1.042	0.001	1.020	0.997-1.044	0.095	1.026	1.002-1.052	0.034
KPS	0.992	0.980-1.004	0.169	0.982	0.964-1.001	0.063	0.996	0.980-1.012	0.628
resection	0.814	0.603-1.099	0.178	0.656	0.373-1.154	0.144	0.935	0.641-1.363	0.727
therapy	0.872	0.800-0.951	0.002	0.835	0.723-0.964	0.014	0.910	0.809-1.024	0.117
MGMT	0.827	0.620-1.103	0.196	0.825	0.511-1.332	0.432	0.812	0.561-1.175	0.269
**SOX10^high^**	1			1			1		
**SOX10^low^**	1.552	1.025-2.352	0.038	2.842	1.241-6.511	0.014	1.144	0.686-1.909	0.605
age	1.024	1.007-1.041	0.005	1.018	0.994-1.043	0.140	1.027	1.001-1.053	0.042
KPS	0.985	0.974-0.996	0.008	0.974	0.957-0.991	0.003	0.992	0.977-1.007	0.288
resection	0.754	0.562-1.011	0.059	0.733	0.438-1.227	0.237	0.835	0.573-1.216	0.347
therapy	0.898	0.824-0.978	0.014	0.851	0.747-0.969	0.015	0.942	0.837-1.060	0.321
MGMT	0.844	0.638-1.116	0.234	0.863	0.575-1.296	0.478	0.826	0.567-1.202	0.317

The reference variable is indicated by HR = 1. The models were adjusted for age, Karnofsky performance scale (KPS), surgical resection, therapy and MGMT methylation status. HR, Hazard Ratio; CI, 95% confidence interval.

The markers tested in these models are indicated in bold font.

**Table 3:** Multivariate Cox regression analysis of p120-catenin and SOX10 in relation to progression-free survival in GBM IDH^wt^ patients.

	All patients			Female patients			Male patients		
	HR	CI	*P*-value	HR	CI	*P*-value	HR	CI	*P*-value
**p120^low^**	1			1			1		
**p120^high^**	2.542	1.499-4.312	<0.001	1.585	0.581-4.320	0.368	2.431	1.222-4.836	0.011
age	1.011	0.989-1.034	0.333	1.040	1.002-1.079	0.039	0.994	0.959-1.031	0.757
KPS	1.001	0.983-1.020	0.902	1.018	0.981-1.057	0.352	0.994	0.972-1.016	0.579
resection	1.047	0.673-1.627	0.839	1.847	0.778-4.385	0.164	1.098	0.627-1.926	0.743
therapy	0.886	0.776-1.012	0.074	0.643	0.510-0.812	<0.001	1.022	0.828-1.261	0.840
MGMT	0.615	0.381-0.970	0.036	0.455	0.185-1.118	0.086	0.625	0.367-1.064	0.083
**SOX10^high^**	1			1			1		
**SOX10l^ow^**	1.553	0.817-2.952	0.180	2.940	0.839-9.305	0.092	0.951	0.426-2.124	0.902
age	1.012	0.989-1.037	0.308	1.038	1.001-1.077	0.044	0.993	0.956-1.032	0.722
KPS	0.997	0.980-1.014	0.692	1.007	0.974-1.041	0.695	0.987	0.966-1.009	0.248
resection	0.893	0.583-1.368	0.603	1.408	0.638-3.108	0.397	0.923	0.537-1.586	0.772
therapy	0.953	0.834-1.088	0.476	0.693	0.562-0.855	<0.001	1.099	0.891-1.355	0.377
MGMT	0.644	0.416-0.998	0.049	0.485	0.214-1.099	0.083	0.638	0.374-1.088	0.099

The reference variable is indicated by HR = 1. The models were adjusted for age, Karnofsky performance scale (KPS), surgical resection, therapy and MGMT methylation status. HR; Hazard Ratio; CI; 95% confidence interval.

The markers tested in these models are indicated in bold font.

## Discussion

In this study, we investigated the expression levels of three biomarker candidates (p120-catenin, COL4A2 and SOX10) in gliomas with different tumor grades, as well as in the adjacent healthy brain tissues. The main part of the study aimed to characterize the prognostic relevance of these markers for the overall- and progression-free survival of patients with GBM IDH^wt^ tumors, with an additional focus on potential sex-related differences of the markers in this type of cancer.

Our results demonstrated that p120-catenin was expressed at low levels in healthy brain tissues, but was gradually upregulated with increasing tumor malignancy, and exhibited the highest expression in GBM IDH^wt^ tissues. These findings are in agreement with two recent studies. Specifically, Gritsenko et al. showed that *p120-catenin* mRNA expression was upregulated in low-grade gliomas compared to normal brain tissue, and increased further in high-grade gliomas (11). Additionally, Wang and colleagues performed comprehensive analyzes of p120-catenin expression in healthy brain tissues and gliomas with different degrees of malignancy, thereby demonstrating a highly significant positive correlation between p120-catenin and tumor grade at both mRNA and protein level (13). The gradual increase in p120-catenin levels observed in the previous and our own studies suggests an involvement of this marker in glioma progression. Indeed, accumulating evidence indicates that p120-catenin can regulate important pro-tumor functions in glioma cells. Studies by Han and co-workers demonstrated that p120-catenin promoted the proliferation, migration and the epithelial-to-mesenchymal transition (EMT) of U87 and U251 glioma cells via the Wnt/β-catenin signaling pathway (12). Another study found that *p120-catenin* knock-down inhibited microtubule formation in glioma cells, and decreased their proliferative, invasive and migratory ability. These cells simultaneously exhibited an increased intracellular concentration of calcium ions, as well as hallmarks of apoptosis (13). Studies from our own group showed that p120-catenin was a downstream target of PLOD2, which was involved in GBM cell proliferation, invasion and anchorage-independent growth, but also in the modulation of the immune microenvironment in these tumors (30). Additionally, comprehensive *in vivo* studies on glioma infiltration into the brain parenchyma identified p120-catenin as a critical regulator of multicellular network formation and the collective migration of the tumor cells. Further transcriptomics analyzes showed that *p120-catenin* was an upstream regulator of neurogenesis and cell cycle pathways in these tumors (11).

Despite its important cellular functions, the role of p120-catenin as a prognostic marker in glioma and GBM remains poorly characterized, since only one other study addressed this issue to date. Using the gene expression database MAS5.0-u133p2, this study found that glioma patients with high *p120-catenin* expression had a significantly shorter overall survival compared to patients with low levels of this marker (11). It is, however, relevant to mention that the authors did not take into account the tumor grade, but instead performed the survival analyzes on a pool of glioma grades 1-4. Furthermore, no multivariate analyzes were performed to determine the prognostic value of p120-catenin in these patients. Our study focused on a more homogenous cohort of only GBM IDH^wt^ patients, and found that the high protein levels of p120-catenin significantly associated with the poor overall and progression-free survival of these patients. Additional multivariate Cox regression analyzes demonstrated that p120-catenin was an independent and potentially valuable prognostic marker in this type of cancer.

In regard to COL4A2, our data revealed a significant inverse correlation between the expression of this marker and the tumor grade, with the lowest levels being observed in high-grade gliomas. Interestingly, previous proteomics studies showed that COL4A2 was downregulated in response to Interleukin-1 (IL-1) in GBM (31), while other groups linked COL4A2 downregulation to hypoxia and hypoxia-inducible factor 1α (HIF1α) (32, 33). Considering that both IL-1 and hypoxia/HIF1α associate with high tumor grade and progression in gliomas (34–38), the decreased expression of COL4A2 observed in our astrocytoma grade 3 and GBM patients may be, at least partially, explained by these mechanisms. Surprisingly however, our findings are in contrast to the previous studies in glioma, which found a positive correlation between the expression of COL4A2 and tumor grade, thereby suggesting a tumor-promoting role of COL4A2 in these tumors (15–17). We do not currently have an explanation for this discrepancy, but it should be pointed out that these studies assessed the gene expression of *COL4A2*, while our analyses focused on the protein levels of this marker. The above-mentioned studies also found an association between high *COL4A2* gene expression and the poor overall survival of the patients (15–17). However, these survival analyses were performed on heterogeneous glioma cohorts, which included both low-grade and high-grade tumors. In contrast, our study on only GBM IDH^wt^ patients, found no significant association between COL4A2 and the patients’ overall- or progression-free survival, regardless of the cut-off. Similar results were obtained by Choi and colleagues, who did not find a prognostic significance for COL4A2 in their entire cohort of GBM patients, but only in a bevacizumab-treated subgroup (39). Thus, it appears that the role of COL4A2 in glioma pathophysiology and prognosis is still unclear, and would benefit from a more extensive characterization in future studies.

Finally, our study demonstrated a markedly decreased expression of SOX10 in glioma tissues compared to healthy adjacent brain tissues, with the lowest levels being observed in high-grade gliomas. These findings are supported by previous studies, which showed -albeit on a very small number of patients- that healthy brain tissues were largely positive for SOX10 protein (2 out of 2 samples), while most GBM tissues contained few or no positive cells (5 out of 6 samples) (26). Further studies on different types of glioma found a significant loss of SOX10 protein in GBM tissues (26-43% positive samples) compared to WHO grade 1 tumors, such as pilocytic astrocytoma (100% positive samples) (40, 41). Taken together, these findings indicate a link between SOX10 downregulation and tumor malignancy in gliomas, and suggest that SOX10 is a tumor-suppressing factor whose loss may facilitate the progression of these tumors. Indeed, recent molecular and functional studies strongly support this hypothesis. For instance, Wu and colleagues demonstrated that *SOX10* loss caused a proneural-mesenchymal subtype switch *in vitro*, and increased tumor invasion, immune infiltration and mortality in an *in vivo* syngeneic graft GBM model (28). In other elegant studies, Man and co-workers very recently showed that *SOX10* knock-down induced the phenotypic plasticity of GBM, which was characterized by an aggressive neural stem cell (NSC)-like phenotype. Importantly, this phenomenon could be neutralized *in vivo* by Notch and HDAC/PI3K inhibitors, thereby providing a rationale for the design of novel therapeutic strategies (27).

The prognostic relevance of SOX10 in GBM has only been addressed by one other study thus far, but the results were not very conclusive. Using the TCGA database, Xiao and colleagues found that low gene expression levels of *SOX10* tended to associate with a shorter overall survival of the GBM patients. However, the opposite was observed in GBM patients from the CGGA database (42). Our survival analyses showed that GBM IDH^wt^ patients with low SOX10 protein expression had a shorter overall- and progression-free survival compared to SOX10^high^ patients. In the multivariate analysis, SOX10 was an independent prognostic factor for the overall survival of these patients. Importantly, more detailed analysis according to the patients’ sex revealed that SOX10 was significantly associated with survival only in female GBM IDH^wt^ patients. Although no molecular or functional evidence is currently available to provide an explanation for this phenomenon, SOX10 may be nevertheless a sensitive and valuable prognostic marker in female patients with GBM IDH^wt^ tumors, since low levels of SOX10 predicted an almost three-fold higher risk of death in these patients. These findings also underscore the importance of sex-dependent evaluation of biomarkers. While such studies are relatively well established in the field of neurodegenerative or cardiovascular research (for recent reviews see (43–46)), they are largely missing in the field of neuro-oncology. However, recent evidence indicates significant sex-related differences in the pathophysiology of brain tumors, including gliomas. Complex multi-omics analyses identified numerous molecular features associated with biological sex in GBM patients (47), including key differences in the MGMT promoter methylation, genomic instability, and EGFR phosphorylation (48). Prosperetti et al. found that EGFR amplification was significantly more frequent in female GBM patients (49), while Spornagel and colleagues identified sex-related differences in the glutamine metabolism of gliomas (50). Additionally, biomarker studies by Xu et al. found that serum miR-4297 was a significant predictor of tumor grade and of progression-free survival in female, but not in male glioma patients (51). Together with our own findings, these studies indicate that future investigations employing sex-stratified analyses may significantly contribute to the improvement of precision medicine in glioma and other brain tumors.

In summary, all three markers analyzed in this study were differentially expressed in glioma tissues compared to healthy brain tissues, and correlated with the degree of tumor malignancy. High levels of p120-catenin significantly associated with a shorter overall- and progression-free survival in GBM IDH^wt^ patients, both male and female. In female GBM IDH^wt^ patients, low levels of SOX10 associated with a shorter overall survival of these patients. Additionally, both p120-catenin and SOX10 were significant independent prognostic factors for the overall survival of the respective groups of patients. While these findings need to be validated on additional patient cohorts and, importantly, take into consideration also the molecular subtypes of gliomas, they provide potentially valuable insight into the pathophysiology and prognosis of these tumors.

## Data Availability

The raw data supporting the conclusions of this article will be made available by the authors, without undue reservation.
